# Reassessment of the Unique Mode of Binding between Angiotensin II Type 1 Receptor and Their Blockers

**DOI:** 10.1371/journal.pone.0079914

**Published:** 2013-11-08

**Authors:** Shin-Ichiro Miura, Naoki Nakao, Hiroyuki Hanzawa, Yoshino Matsuo, Keijiro Saku, Sadashiva S. Karnik

**Affiliations:** 1 Department of Cardiology, Fukuoka University School of Medicine, Fukuoka City, Fukuoka, Japan; 2 Department of Molecular Cardiovascular Therapeutics, Fukuoka University School of Medicine, Fukuoka City, Fukuoka, Japan; 3 Department of Molecular Cardiology, Cleveland Clinic Foundation, Cleveland, Ohio, United States of America; 4 R&D Division, Daiichi Sankyo Company, Ltd., Tokyo, Japan; Medical School of Hannover, Germany

## Abstract

While the molecular structures of angiotensin II (Ang II) type 1 (AT_1_) receptor blockers (ARBs) are very similar, they are also slightly different. Although each ARB has been shown to exhibit a unique mode of binding to AT_1_ receptor, different positions of the AT_1_ receptor have been analyzed and computational modeling has been performed using different crystal structures for the receptor as a template and different kinds of software. Therefore, we systematically analyzed the critical positions of the AT_1_ receptor, Tyr^113^, Tyr^184^, Lys^199^, His^256^ and Gln^257^ using a mutagenesis study, and subsequently performed computational modeling of the binding of ARBs to AT_1_ receptor using CXCR4 receptor as a new template and a single version of software. The interactions between Tyr^113^ in the AT_1_ receptor and the hydroxyl group of olmesartan, between Lys^199^ and carboxyl or tetrazole groups, and between His^256^ or Gln^257^ and the tetrazole group were studied. The common structure, a tetrazole group, of most ARBs similarly bind to Lys^199^, His^256^ and Gln^257^ of AT_1_ receptor. Lys^199^ in the AT_1_ receptor binds to the carboxyl group of EXP3174, candesartan and azilsartan, whereas oxygen in the amidecarbonyl group of valsartan may bind to Lys^199^. The benzimidazole portion of telmisartan may bind to a lipophilic pocket that includes Tyr^113^. On the other hand, the n-butyl group of irbesartan may bind to Tyr^113^. In conclusion, we confirmed that the slightly different structures of ARBs may be critical for binding to AT_1_ receptor and for the formation of unique modes of binding.

## Introduction

Angiotensin II (Ang II) type 1 (AT_1_) receptor is a member of the G protein-coupled receptor (GPCR) superfamily and contains 359 amino acids [1]. It has a widespread tissue distribution and mediates most known cardiovascular functions including vasoconstriction, cardiovascular hypertrophy and hyperplasia [2]. AT_1_ receptor blockers (ARBs, including EXP3174, which is an active metabolite of losartan, candesartan, eprosartan, valsartan, telmisartan, olmesartan, irbesartan, and azilsartan) have been developed and are available for clinical use worldwide. Basic and clinical studies have shown that ARBs are useful for preventing the development of cardiovascular disease [2].

With the exception of eprosartan, ARBs that are widely used in clinics share a common molecular scaffold consisting of biphenyl-tetrazol and imidazole groups that have slightly different structures [3,4]. Recent clinical studies have demonstrated that not all ARBs have the same effects and some benefits conferred by ARBs may not be class (or common) effects, but rather molecule-specific (or differential) effects [4]. We and others previously indicated that each ARB has a unique mode of binding for AT_1_ receptor [5-16]. We have proposed that the molecule-specific effects may be due to small differences in the molecular structure of each ARB [3].

While the crystal structures of GPCRs obtained from the rhodopsin, opsin, and beta_1_- and beta_2_-adrenergic receptor systems have recently been described [17-24], the crystal structure of AT_1_ receptor has not been elucidated. Although we and others analyzed the mode of binding of ARBs to AT_1_ receptor [5-16], different approaches used showed different modes of binding of ARBs to AT_1_ receptor. These analyses considered different positions of the AT_1_ receptor using site-directed mutagenesis and performed computational modeling using different GPCR crystal structures as templates and also different softwares which could account for the different modes of binding of ARBs to AT_1_ receptor observed.

To resolve these problems, in this study we systematically analyzed the same critical positions of AT_1_ receptor, Tyr^113^, Tyr^184^, Lys^199^, His^256^ and Gln^257^, which may commonly bind to ARBs according to previous reports [5-16], using a mutagenesis study, and subsequently performed computational modeling of the binding mode between AT_1_ receptor and ARBs using human C-X-C chemokine receptor type 4 (CXCR4) receptor as a new template [25] and a single version of software. We confirmed here that the slightly different structures of ARBs are critical for unique modes of binding to AT_1_ receptor.

## Materials and Methods

### Materials

**Figure 1:**
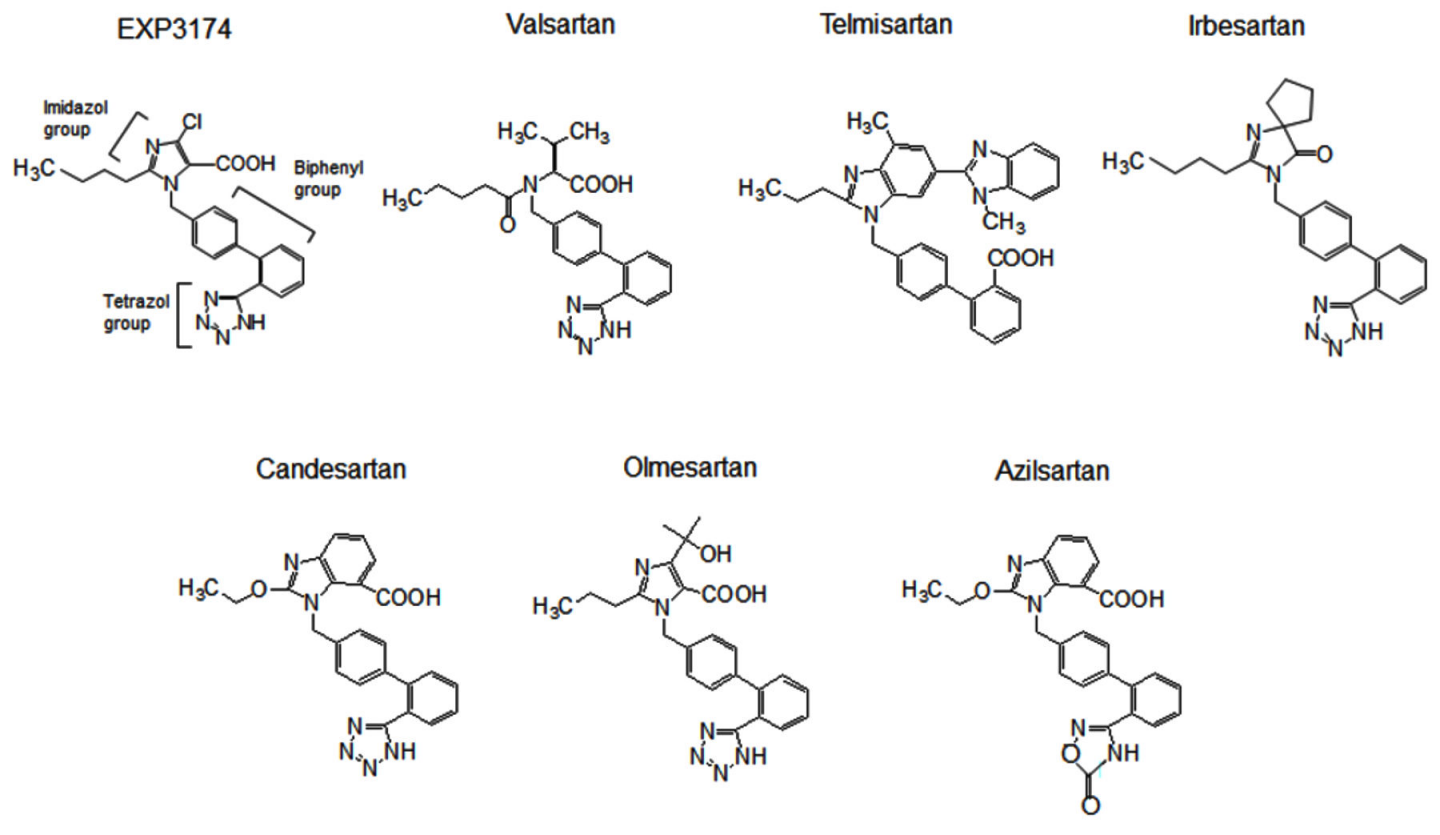
Chemical structures of the angiotensin II type 1 receptor blockers EXP3174, which is an active metabolite of losartan, candesartan, valsartan, telmisartan, olmesartan, irbesartan and azilsartan.

The following reagents were purchased or provided: EXP3174, candesartan, valsartan, telmisartan, irbesartan and azilsartan (Toronto Research Chemicals Inc., Ontario, Canada); olmesartan (Daiichi Sankyo Co., Tokyo, Japan); [Sar^1^, Ile^8^]Ang II (Sigma-Aldrich, MO, USA); and ^125^I-[Sar^1^, Ile^8^] Ang II (Amersham Biosciences, Buckinghamshire, UK). The molecular structures of the ARBs are shown in Figure 1.

### Site-directed mutagenesis and expression of the AT_1_ receptor and membrane preparation

The synthetic rat AT_1_-wild-type (WT) receptor gene, cloned in the shuttle expression vector pMT-3, was used for expression and site-directed mutagenesis studies, as described previously [26].

### Cell cultures, transfections, membrane preparation

COS1 cells (African green monkey kidney fibroblast-like cell line, #CRL-1650, American Type Culture Collection, VA, USA) were cultured. The cells were maintained in 10 % fetal bovine serum and penicillin- and streptomycin-supplemented Dulbecco’s modified Eagle’s essential medium (Invitrogen) in 5 % CO_2_ at 37°C. In the experiments, cells without cell-growth supplement were used. The WT and mutant AT_1_ receptors were transiently transfected into COS1 cells using Lipofectamine 2000 liposomal reagent (Roche Applied Science) according to the manufacturer’s instructions. Cell membranes were prepared by the nitrogen Parr bomb disruption method using a 0.25 M sucrose solution in the presence of protease inhibitors.

### Competition binding study

The K_d_ values of receptor binding were determined by ^125^I-[Sar^1^, Ile^8^]Ang II-binding experiments under equilibrium conditions, as described previously [26]. Membranes expressing the AT_1_-WT or mutant receptors were incubated with 100 pM ^125^I-[Sar^1^, Ile^8^], [Sar^1^, Ile^8^]Ang II or ARBs for 1 h at 22 °C in a volume of 125 μl. The binding reaction was terminated by filtering the incubation mixture through Whatman GF/C glass filters, and the residues were extensively washed further with binding buffer. The bound ligand fraction was determined from the counts per minute (cpm) remaining on the membrane. Binding kinetics values were determined as previously described [26].

### Molecular Modeling of the AT_1_ Receptor

Unless otherwise noted, all analyses were performed using Prime, version 3.0 (Schrödinger, LLC, New York, NY, 2011). The X-ray crystal structure of human CXCR4 (Protein Data Bank code 3OE0) was used as the template for the AT_1_ receptor model. The primary sequence of AT_1_ receptor was obtained from the Swiss-Prot Protein Database (AGTR1_HUMAN, P30556) and aligned with that of the CXCR4 structure by considering the sequence identity, secondary structure prediction results and typical consensus sequence of GPCRs (disulfide bond between transmembrane (TM) 3 and extracellular loop (ECL) 2, LAxAD in TM2, DRY in TM3, [F/Y]xxPxxxxxxxY in TM5 and (C)WxP in TM6). Using this alignment, we built a comparative homology model of the AT_1_ receptor from the X-ray structure of CXCR4. Ligand and the T4-Lysozyme region (1102-1161) in the CXCR4 structure were removed before modeling. The protonation state of His^256^ of the model was modified manually to the epsilon position. The final AT_1_ receptor model had 289 amino acids (15-303) and 31 % sequence identity with the template. The stereochemical quality of the model was assessed by PROCHECK analysis [27].

### Induced fit docking (IFD) of olmesartan

The ligand 3D structure of olmesartan was generated by LigPrep version 2.5 (Smchrödinger, LLC, New York, NY, 2011). The ligand was docked into the AT_1_ receptor model using the Schrödinger Suite 2011 IFD protocol. The VDW scaling factors for both the ligand and the protein were set to 0.3 and Lys^199^ in TM5 was specified as the ligand binding site. All other parameters were at the default setting.

Thirteen models were obtained as a result of induced fit docking. The interaction fingerprints [28] were calculated for the models by using the Python script bundled in Schrödinger Suite 2011. Using this information, we chose the best model that satisfied polar interactions between the ligand and all four of the important residues (Tyr^113^, Lys^199^, His^256^, Gln^257^) in the AT_1_ receptor suggested by the mutation study. Energy minimization was performed with the model using MacroModel, version 9.9 (Schrödinger, LLC, New York, NY, 2011). The force field was set to OPLS2005 with the GB/SA water solvation model and all atoms of the protein backbone were fixed during minimization. The final AT_1_ receptor model with olmesartan was obtained by the Polak-Ribier Conjugate Gradient method with a convergence threshold of 0.005 for less than 5000 iterations. We used the AT_1_ receptor model as the docking template for the various ARBs as described below.

### Docking of various ARBs to the AT_1_ receptor model

The 7 ARBs (candesartan, EXP3174, irbesartan, olmesartan, telmisartan, valsartan, azilsartan) were docked into the AT_1_ receptor model by using Glide, version 5.7 (Schrödinger, LLC, New York, NY, 2011) with the hydrogen bond constraint to Lys^199^. Extra precision mode was used for the docking studies, except the irbesartan. Standard precision mode was used for irbesartan since no poses were obtained with the XP mode. The docking poses that had the best glide score for each ARB were selected as the final model.

## Results

### Binding affinities of [Sar^1^, Ile^8^]Ang II and ARBs to AT_1_-WT and mutant receptors

First, we selected the candidate residues (Tyr^113^, Tyr^184^, Lys^199^, His^256^ and Gln^257^) for specific binding sites of ARBs based on the molecular models of the AT_1_ receptor complex described in previous reports [5-16]. As shown in Table S1, the binding affinities of [Sar^1^, Ile^8^]Ang II and ARBs to AT_1_-WT and mutant receptors were performed in the present study and our previous studies [8-10,12,16]. The ratio of K_d_ (mutant) to K_d_ (WT) [K_d_ (mutant)/K_d_ (WT)] were calculated.

First, the binding affinities of [Sar^1^, Ile^8^]Ang II and ARBs to AT_1_-WT were performed, and we confirmed that the data of binding affinities in the present study and our previous studies [8-10,12,16] were similar. The affinities of [Sar^1^, Ile^8^]Ang II were almost the same in some mutants and were decreased in other mutants, but not to less than 1/5 the affinity for the AT_1_-WT receptor.

The affinity of EXP3174 was reduced by more than 10-fold in Y113A, K199A and Q257A mutant receptors compared to the AT_1_-WT receptor, suggesting that Tyr^113^, Lys^199^ and Gln^257^ in the AT_1_ receptor are involved in binding to EXP3174. Similar to EXP3174, valsartan, candesartan and azilsartan showed a more than 10-fold loss of affinity in Y113A, K199A and Q257A mutant receptors compared to the AT_1_-WT receptor, which indicated that Tyr^113^, Lys^199^ and Gln^257^ in the AT_1_ receptor are also involved in binding to the 4 ARBs which have a carboxyl group as a common chemical structure.

Telmisartan (13-fold reduction) and irbesartan (27-fold reduction) only exhibited a >10-fold reduction in binding affinity to the Y113A mutant receptor. These ARBs retained their binding affinities toward other mutant receptors (<10-fold reduction). Telmisartan and irbesartan do not contain a carboxyl group and instead have a benzimidazole portion and a cyclopentyl group, respectively.

Among the remaining ARBs, olmesartan, which contains a hydroxyl group in addition to a carboxyl group, showed a >10-fold reduction in binding affinity to the Y113F (23-fold reduction), K199A (60-fold reduction), K199Q (14-fold reduction), H256A (16-fold reduction), and Q257A (98-fold reduction) mutant receptors. Olmesartan may bind to Tyr^113^, Lys^199^, His^256^ and Gln^257^ in the AT_1_ receptor.

### Binding affinities of telmisartan to other AT_1_ mutant receptors

Ohno et al. reported that all of the ARBs interacted with Phe^171^, Phe^182^, Tyr^184^, Lys^199^ and His^256^, and telmisartan possessed unique additional interaction sites that could bind to hydrophobic residues (Val^116^, Phe^204^, Phe^208^ and Trp^253^) [14]. Since they indicated that the binding mode of telmisartan is much different than those of other ARBs, we performed an additional site-directed mutagenesis study. At these positions in the AT_1_ receptor, we constructed AT_1_ mutant receptors, i.e., V116A, F182A, Y184A, F208A and W253A (Table S1). The affinities of [Sar^1^, Ile^8^]Ang II were almost the same in the mutants (<3-fold reduction). The positions Val^116^, Phe^182^, Tyr^184^, Phe^208^ and Trp^253^ are all not important for binding to telmisartan because telmisartan retained its binding affinity against the mutant receptors, i.e., V116A, F182A, Y184A, F208A and W253A (<2-fold reduction).

### Molecular models of the interactions between ARBs and the AT_1_ receptor

**Figure 2:**
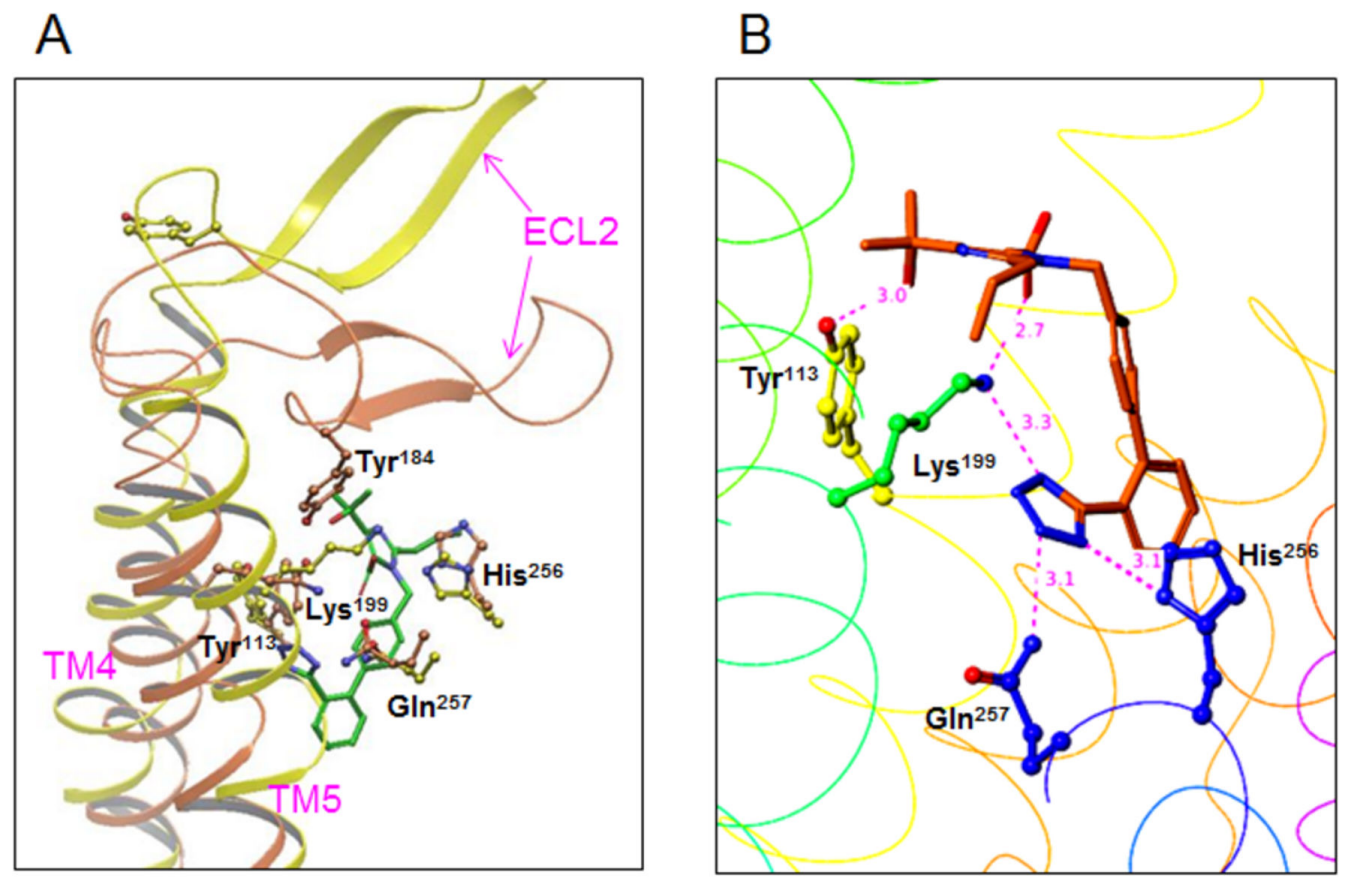
A. Position of the second extracellular loop (ECL2) in the model of AT_1_ receptor using bovine rhodopsin (orange) or CXCR4 (yellow) receptor as a template. B. Molecular modeling of the interaction between olmesartan and the AT_1_ receptor. The AT_1_ receptor is shown as a ribbon and Tyr^113^, Lys^199^, His^256^, Gln^257^ and olmesartan are shown as stick models. Color notation in the helix is as follows: transmembrane (TM)1, orange; TM3, yellow; TM5, dark green; TM6, blue; and TM7, purple.

Next, molecular modeling of the binding modes of AT_1_ receptor and ARBs was performed. Previous studies performed computational modeling using different crystal structures of the receptor as a template, mainly bovine rhodopsin [5-16]. We used the recently published structure of the CXCR4 receptor as a new template [25]. The position of ECL2 in the model of AT_1_ receptor with bovine rhodopsin as a template was much different from the position of Tyr^184^ with CXCR4 receptor (Figure 2A). The position of TM5 in the AT_1_ receptor was also different.

For modeling, IFD was performed using the AT_1_ receptor model and olmesartan among the 7 kinds of ARBs. Since there are a lot of different modes of binding between olmesartan and AT_1_ receptor, it is generally difficult to classify them. A couple of binding modes were selected among the different modes, since olmesartan binds to 4 sites in the AT_1_ receptor (Tyr^113^, Lys^199^, His^256^ and Gln^257^) as suggested from the mutation experiment. Among a couple of modes, the mode of binding that showed the highest IFD score was selected (Figure 2B). The interactions between Tyr^113^ in the AT_1_ receptor and the hydroxyl group of olmesartan (distance of 3.0 Å), between Lys^199^ and the carboxyl (2.7 Å) or tetrazole (3.3 Å) group, and between His^256^ (3.1 Å) or Gln^257^ (3.1 Å) and the tetrazole group were examined. These are reasonable distances for contributing to electrostatic and/or hydrogen bond interactions.

**Figure 3:**
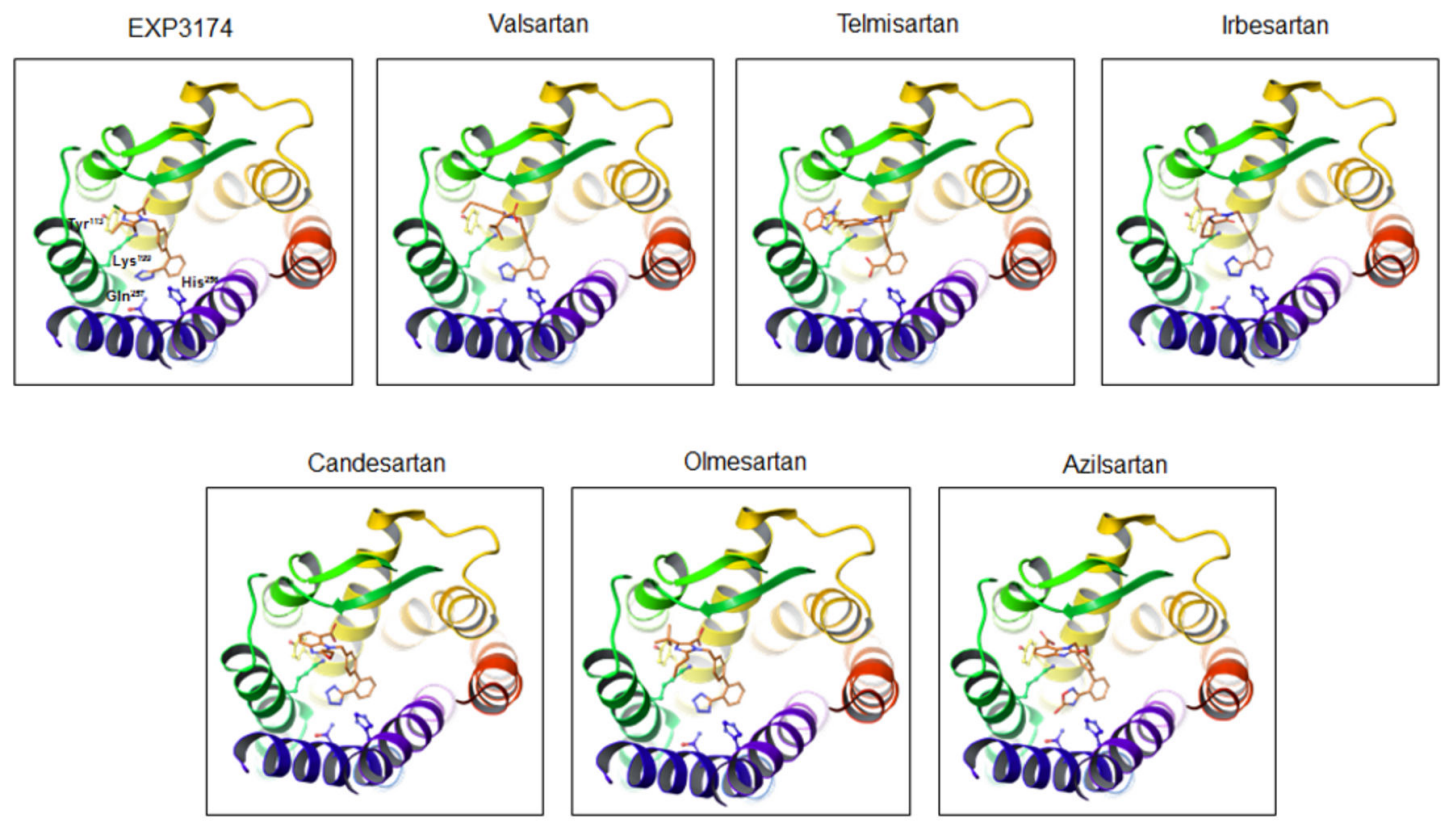
Molecular modeling of the interactions between the 7 ARBs and the AT_1_ receptor. Color notation in the helix is as follows: transmembrane (TM)1, orange; TM2, dark yellow; TM3, yellow; TM4, green; TM5, dark green; TM6, blue; and TM7, purple.

**Figure 4:**
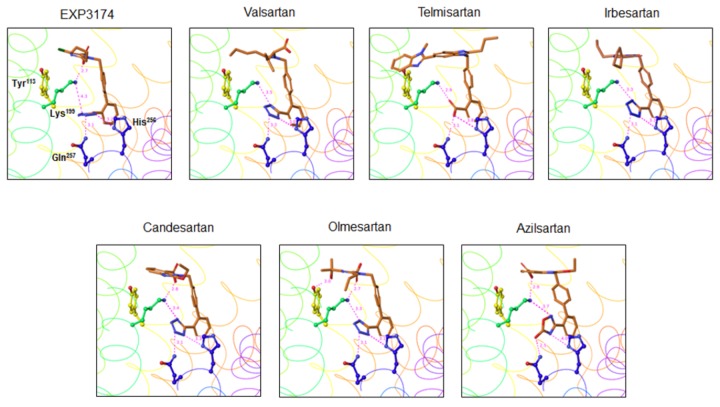
Molecular modeling of a close-up view of the interactions between the 7 ARBs and the AT_1_ receptor. Color notation in the helix is as follows: transmembrane (TM)1, orange; TM3, yellow; TM5, dark green; TM6, blue; and TM7, purple.

As shown in Figures 3 and 4, we also performed molecular modeling of the modes of binding between the remaining 6 ARBs and AT_1_ receptor based on the mode of binding between olmesartan and AT_1_ receptor (Figure 2B). A tetrazole group, which is a common chemical structure of ARBs, binds similarly to Lys^199^, His^256^ and Gln^257^ in the AT_1_ receptor. This tetrazole group is replaced by a carboxyl group and 5-oxo-1,2,4-oxadiazole in telmisartan and azilsartan, respectively. Lys^199^ in the AT_1_ receptor binds to the carboxyl groups in EXP3174, candesartan and azilsartan. Although valsartan also contains a carboxyl group, this carboxyl group did not interact with Lys^199^ and may bind to Ser^105^ in the AT_1_ receptor. Instead, oxygen of the amidecarbonyl group of valsartan may bind to Lys^199^.

Telmisartan and irbesartan do not contain a carboxyl group, and instead have a benzimidazole portion and a cyclopentyl group, respectively. The benzimidazole portion of telmisartan may bind to a lipophilic pocket that includes Tyr^113^ in the AT_1_ receptor. On the other hand, the n-butyl group of irbesartan may bind to Tyr^113^ according to the present modeling, although the cyclopentyl group of irbesartan should interact with the lipophilic pocket includes Tyr^113^ [29]. Instead, the cyclopentyl group of irbesartan may bind to Phe^182^, Leu^195^ and Lys^199^.

## Discussion

In the present study, we confirmed that the slightly different structures of ARBs are important for unique modes of binding to AT_1_ receptor using mutagenesis-guided docking to AT_1_ receptor model based on CXCR4 receptor [25] as a new template and a single version of software. Although the tetrazole group (which is replaced by a carboxyl group in telmisartan and a 5-oxo-1,2,4-oxadiazole group in azilsartan) in ARBs commonly binds to Lys^199^, His^256^ and Gln^257^ in the AT_1_ receptor, distinctly different modifications of the biphenyl-tetrazolyl-imidazole scaffold lead to different modes of binding observed.

There are several reasons why we used CXCR4 receptor as a template. First, AT_1_ receptor and CXCR4 receptor show about 34 % homology, which is much higher than that between AT_1_ receptor and bovine rhodopsin (about 20 %), which has been used in most previous studies [5-16]. Second, in the GPCR evolution tree [30,31], the AT_1_ receptor is closer to the CXCR4 receptor, but far from rhodopsin. In this study, although we systematically analyzed critical positions of the AT_1_ receptor, i.e., Tyr^113^, Tyr^184^, Lys^199^, His^256^ and Gln^257^, the position of Tyr^184^ in ECL2 with bovine rhodopsin as a template was much different than the position of Tyr^184^ in the AT_1_ receptor using CXCR4 receptor as a template. The position of Tyr^184^ with CXCR4 receptor as a template is far from the ligand binding pocket of AT_1_ receptor. Although ECL2 is critical for receptor activation when Ang II binds to and changes the conformation of ECL2 [32,33], none of the ARBs can bind to Tyr^184^ in ECL2. In fact, none of the ARBs showed a loss of binding affinity in the Y184A mutant AT_1_ receptor in our study.

The main purpose of the present study was to confirm that the slightly different structures of ARBs may be important for binding to AT_1_ receptor and for the formation of unique modes of binding, although a tetrazole group in ARBs commonly binds to Lys^199^, His^256^ and Gln^257^ in the AT_1_ receptor. Our current understanding is that the hydroxyl and carboxyl groups of olmesartan interact with Tyr^113^ and Lys^199^, respectively. We previously indicated that the inverse agonist activity of olmesartan required two interactions that between the hydroxyl group of olmesartan and Tyr^113^ in the receptor and that between the carboxyl group and Lys^199^ and His^256^ in the receptor [8]. We confirmed that the hydroxyl and carboxyl groups in olmesartan were critical for binding. On the other hand, the carboxyl groups of EXP3174, candesartan and azilsartan bind to Lys^199^; these three ARBs do not contain a hydroxyl group and may not interact with Tyr^113^. In addition, valsartan also contains a carboxyl group, which does not bind to Lys^199^. Instead, oxygen of the amidecarbonyl group and the carboxyl group of valsartan may bind to Lys^199^ and Ser^105^, respectively, since we had reported that Ser^105^ is a candidate for binding to the carboxyl group of valsartan [9]. Thus, each ARB showed a slightly different mode of binding with regard to Tyr^113^ and Lys^199^ in the AT_1_ receptor.

**Figure 5:**
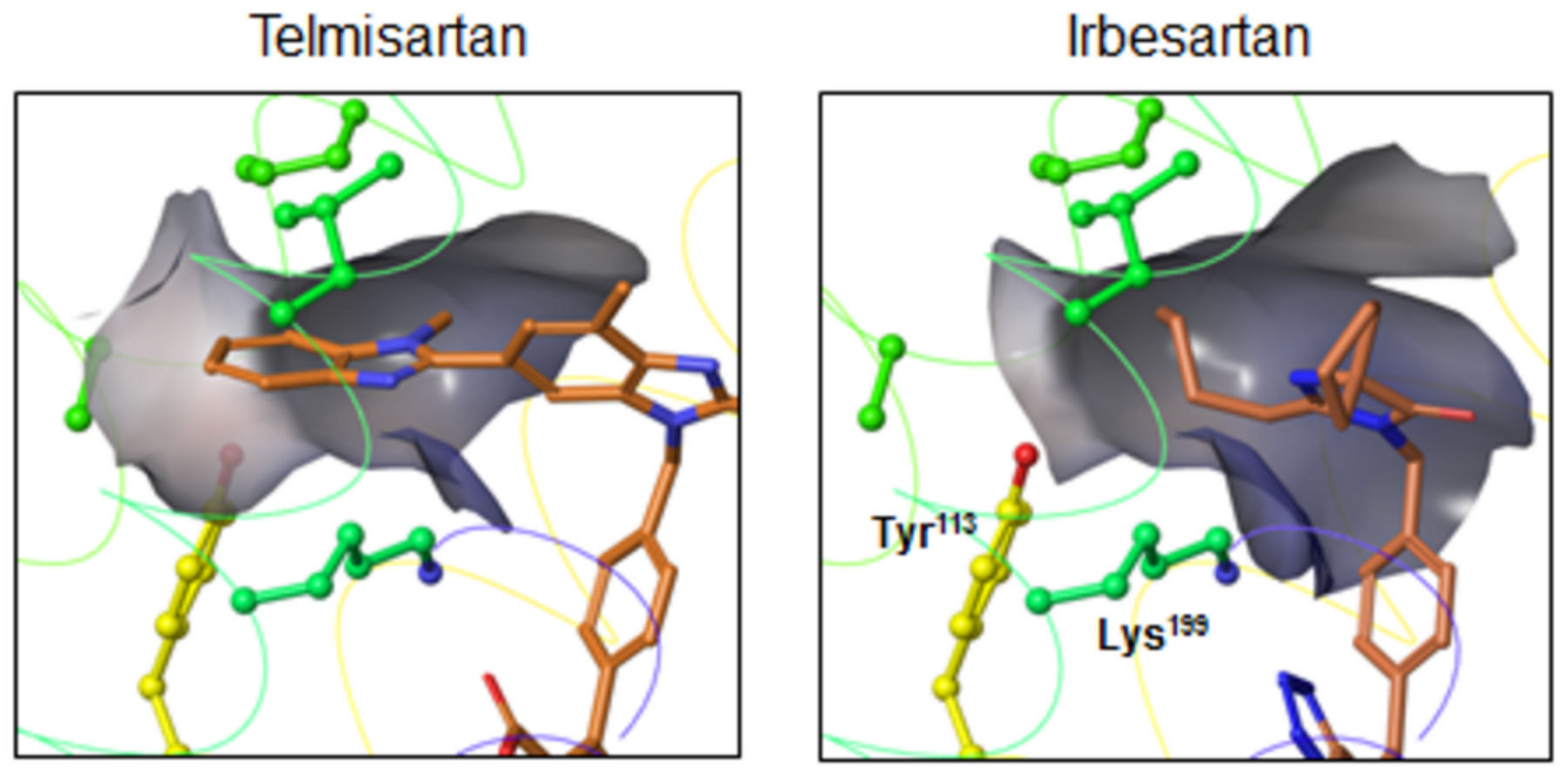
Possible molecular modeling of the interactions between a lipophilic pocket (gray zone) in the AT_1_ receptor and telmisartan or irbesartan. Color notation in the helix is as follows: transmembrane (TM)3, yellow; TM5, dark green; and TM6, blue.

Since telmisartan and irbesartan do not contain an imidazole ring with a carboxyl group, these ARBs should be considered separately from the other ARBs which do contain a carboxyl group. According to results of modeling, the benzimidazole portion of telmisartan may bind to a lipophilic pocket that includes Tyr^113^ by the hydrophobic effect, such as van der Waals forces (Figure 5). Ohno et al. also reported that the benzimidazole portion of telmisartan may interact with the lipophilic pocket in a model of the AT_1_ receptor [14]. Our site-directed mutagenesis study indicated that Val^116^, Phe^182^, Tyr^184^, Phe^208^ and Trp^253^ in addition to Lys^199^, His^256^ and Gln^257^ are not important for binding to telmisartan. In contrast, the data suggest that the benzimidazole portion of telmisartan may be more important for binding to the lipophilic pocket of AT_1_ receptor. The present results showed that the n-butyl group of irbesartan may bind to Tyr^113^ (Figure 5). We previously reported that the phenyl group at Tyr^113^ in the AT_1_ receptor and the cyclopentyl group of irbesartan may exhibit hydrophobic interaction [12]. In fact, the cyclopentyl group should move toward Tyr^113^ to slightly move the protein side chains, but because the chains are fixed in the docking studies, steric interference may occur and thus the correct original position cannot be obtained in the present modeling.

In conclusion, we confirmed that the slightly different structures of ARBs may be critical for binding to AT_1_ receptor and for the formation of unique modes of binding.

## Supporting Information

Table S1 **Binding affinities (K_d_, nM) of [Sar^1^, Ile^8^]Ang II and ARBs to AT_1_ WT and mutants receptors.**
(DOCX)
