# Supplementary material for: Reassessment of the Unique Mode of Binding between Angiotensin II Type 1 Receptor and Their Blockers
Source: PLoS One. 2013 Nov 8;8(11):e79914. doi: 10.1371/journal.pone.0079914 (PMC3832659; doi:10.1371/journal.pone.0079914)
Supplement: Table S1 — Binding affinities (Kd, nM) of [Sar1, Ile8]Ang II and ARBs to AT1 WT and mutants receptors. (DOCX) [file pone.0079914.s001.docx]

Supplementary Table 1. Binding affinities (K_d_, nM) of [Sar^1^, Ile^8^]Ang II and ARBs to AT_1_ WT and mutants receptors.

Receptor [Sar^1^, Ile^8^]AngII EXP3174 Valsartan Candesartan Telmisartan Irbesartan Olmesartan Azilsartan

WT 0.8 ± 0.4 (1.0) 3.9 ± 1.5 (1.0) 2.4 ± 0.7 (1.0) 2.3 ± 1.5 (1.0) 5.2 ± 1.8 (1.0) 1.8 ± 0.9 (1.0) 2.3 ± 0.8 (1.0) 3.0 ± 0.2 (1.0)

Y113A 1.0 ± 0.4 (1.3) 358 ± 61 (92) 1704 ± 421 (710) 173 ± 45 (75) 66 ± 14 (13) 49 ± 5 (27) 9.3 ± 2.7 (4.0) 232 ± 96 (77)

Y113F 1.8 ± 1.0 (2.3) 12 ± 3 (3.1) 9.7 ± 2.1 (4.0) 7.9 ± 3.6 (3.4) 8.1 ± 2.7 (1.6) 4.6 ± 0.7 (2.6) 52 ± 7 (23) 14 ± 0.3 (4.7)

V116A 1.0 ± 0.4 (1.3) N.D. N.D. N.D. 6.9 ± 0.9 (1.3) N.D. N.D. N.D.

F182A 0.6 ± 0.2 (0.8) N.D. N.D. N.D. 1.9 ± 0.2 (0.4) N.D. N.D. N.D.

Y184A 1.0 ± 0.4 (1.3) 2.8 ± 0.6 (0.7) 8.3 ± 2.3 (3.5) 2.0 ± 0.7 (0.9) 2.0 ± 0.4 (0.4) 2.1 ± 1.1 (1.2) 5.7 ± 0.6 (2.5) 4.8 ± 1.3 (1.6)

K199A 3.3 ± 0.5 (4.1) 81 ± 11 (21) 434 ± 107 (181) 53 ± 5 (23) 35 ± 6 (6.7) 6.7 ± 0.3 (3.7) 138 ± 9 (60) 103 ± 7 (34)

K199Q 0.7 ± 0.1 (0.9) 18 ± 9 (4.6) 118 ± 15 (49) 14 ± 1 (6.1) 5.3 ± 2.8 (1.0) 2.0 ± 1.0 (1.1) 33 ± 6 (14) 27 ± 5 (9.0)

F208A 0.7 ± 0.3 (0.9) N.D. N.D. N.D. 4.7 ± 1.7 (0.9) N.D. N.D. N.D.

W253A 2.2 ± 0.8 (2.8) N.D. N.D. N.D. 7.9 ± 1.0 (1.5) N.D. N.D. N.D.

H256A 0.8 ± 0.3 (1.0) 6.7 ± 3.1 (1.7) 2.1 ± 1.1 (0.9) 4.3 ± 1.6 (1.9) 33 ± 11 (6.3) 2.4 ± 1.4 (1.3) 36 ± 4 (16) 5.6 ± 2.4 (1.9)

Q257A 2.5 ± 0.2 (3.1) 62 ± 37 (16) 25 ± 5 (10) 37 ± 8 (16) 30 ± 12 (5.8) 3.2 ± 0.7 (1.8) 226 ± 4 (98) 309 ± 22 (103)

Data of binding affinities with underlines and non-underlines were performed in our previous studies [8, 10, 11, 13, 17] and the present study, respectively.

Numbers in parentheses calculated the ratio of Kd (mutant) to Kd (wild type) [Kd (mutant)/Kd (wild type)]. N.D., not determined.
